# Dimethylamine Borane: Tsan and Hung Respond

**DOI:** 10.1289/ehp.114-a274b

**Published:** 2006-05

**Authors:** Yu-Tse Tsan, Dong-Zong Hung

**Affiliations:** Taichung Veterans General Hospital, Taichung, Taiwan, E-mail: hdz66@vghtc.gov.tw

The magnetic resonance imaging (MRI) studies of our patient (Tsan et al. 2005) were performed on the 8th and 37th days after DMAB poisoning and show marked differences. The lesions of the bilateral cerebellar periventricular area were revealed in every mode of images (fluid-attenuated inversion recovery, T2-weighted intensity, diffusion-weighted images, and T1WI) and were comparable with the patient’s symptoms and signs. The possibility of artifact is small. The changes in the serial MRI suggest a transient brain lesion, which may be due to transient demyelination, neuronal damage, or edema.

We appreciate that Kuo et al. (in press) performed the nerve biopsy to prove the axonal polyneuropathy. Their result was comparable with those of our study: DMAB intoxication can lead to acute cortical, cerebellar lesions and polyneuropathy.
